# Fast and accurate visual acuity prediction based on optical aberrations and machine learning

**DOI:** 10.1038/s41598-025-27972-0

**Published:** 2025-12-19

**Authors:** A. Sierra, I. Baoud-Ould-Haddi, S. Fernández-Núñez, J. A. Gómez-Pedrero, M. García-Montero, N. Garzón, J. Alonso, E. Pascual, J. Vargas

**Affiliations:** 1 Departamento de Óptica, Facultad de Ciencias Físicas, Universidad Complutense de Madrid, Plaza de Ciencias 1, 28040 Madrid, Spain; 2 Departamento de Optometría y Visión, Facultad de Óptica y Optometría, Universidad Complutense de Madrid, C. de Arcos de Jalón, 118, 28037 Madrid, Spain; 3 Departamento de Óptica, Facultad de Óptica y Optometría, Universidad Complutense de Madrid, C. de Arcos de Jalón, 118, 28037 Madrid, Spain; 4 Clinical Research Department, Indizen Optical Technologies, 28002 Madrid, Spain

**Keywords:** Visual acuity (VA), Zernike coefficients, Regression trees, XGBoost, Neural networks, Amplitude of accommodation, Optics and photonics, Physics, Biomedical engineering

## Abstract

In this work, we propose three machine learning-based methods for predicting visual acuity (VA). Two methods utilize regression trees (LSBoost and XGBoost), and the third employs a neural network that classifies simulated aberrated optotypes as “recognized” or “unrecognized”. The overall VA is estimated by replicating the clinical procedure in which the subject reads optotypes and the VA is determined based on their responses. Here, the neural network acts as a substitute for the subject. Data were collected from a clinical trial involving 135 subjects providing for each sample 36 Zernike coefficients, amplitudes of accommodation, age, and VA values. Evaluation of the regression tree models demonstrates that LSBoost outperformes XGBoost in prediction accuracy, especially when incorporating amplitudes of accommodation. However, XGBoost is faster in computation time, making it more suitable for large datasets and design of visual compensations. The neural network, while achieving high optotype recognition accuracy, is less accurate in VA prediction due to its reliance on synthetic data and complex simulation processes, which requires large processing times. Overall, LSBoost offers the best performance in terms of accuracy, while XGBoost provides faster computation. These findings highlight the suitability of regression tree-based models for VA prediction using tabulated data.

**Supplementary Information:**

The online version contains supplementary material available at 10.1038/s41598-025-27972-0.

## Introduction

Visual acuity (VA) is one of the fundamental metrics used to assess the quality of an individual’s vision^1^. In essence, VA integrates the three main aspects of human vision, eye resolution power, retinal sampling and neural processing, into a single metric directly related to the minimum angle of resolution (MAR), which corresponds to the smallest angular extent of letters and symbols a person can recognize. It is routinely assessed during visual examinations under various conditions: monocularly or binocularly, at far, intermediate or near distances, and with or without the best optical correction. These measurements provide critical insights into the status of a patient’s vision. In this paper, we will focus on monocular far distance visual acuity so that the binocular summation that occurs with both eyes open does not influence the outcome.

Modelling VA is a complex but rewarding endeavor with applications in diverse fields such as optical design^2^, ergonomics^3^, virtual and augmented reality^4^, medical diagnostics^5^, display technology^6^, and human-machine interfaces. For instance, in the design of visual compensation elements such as ophthalmic lenses, contact lenses, or intraocular lenses (IOLs), individualized VA models are essential to predict and optimize the visual outcomes for each patient. However, modelling VA is inherently challenging due to the multitude of factors influencing an individual’s visual performance. VA can vary significantly between individuals because of differences in ametropia, age, or diseases that impair vision despite optimal optical correction. In healthy individuals, VA is primarily determined by refractive error, accommodation, and pupil size^1^. However, other factors, such as ocular aberrations, the transparency of ocular media, and neurological processing, also contribute. These interdependencies highlight the complexity of accurately modelling VA and underscore the importance of advancing our understanding of this metric.

VA models can be categorized into two main groups: phenomenological and functional. The first group comprises models that empirically derive VA from clinical data obtained from specific populations^7–10^. In 1981, Legge et al.^7^ introduced an inverse power law model describing the relationship between decimal VA and defocus (spherical blur) based on experiments conducted on patients with dilated pupils and paralyzed accommodation. Building on this, Smith^8^ revisited Legge’s work and other studies to propose a more sophisticated function linking decimal VA to the product of refractive error (blur) and pupil diameter. Smith’s model asymptotically converged to an inverse square law for high levels of blur. Raasch^9^ later refined Smith’s results by incorporating astigmatic defocus while neglecting the effect of pupil size. Gomez-Pedrero et al.^10^ further advanced phenomenological modelling by accounting for astigmatic blur, pupil diameter, and the accommodative response. Their model relied on the accommodative compensation for hyperopic refractive errors, explaining the asymmetry in the rate of VA decline with increasing blur between myopic and hyperopic subjects^7^. However, phenomenological models have significant limitations. At best, they consider only primary factors such as refractive errors and pupil diameter, while neglecting other influences like higher-order aberrations and neural processing. Additionally, these models heavily depend on the quality of the data used for parameter fitting. In many cases, such data are collected from uncontrolled experiments, such as vision screenings conducted on groups of military recruits^11^.

Functional (or template) VA models^12–14^ aim to compute VA from the wavefront aberration of the subject by mimicking the human vision process. These models generally follow a four-step framework: optical processing, neural processing, template matching, and VA determination.

1. Optical Processing: This step involves computing the degraded image of an optotype based on the eye’s optical properties. The process begins with calculating the point spread function (PSF) of the eye using the wavefront aberration coefficients of the subject. The optotype image is then convolved with this PSF to produce the aberrated image.
2. Neural Processing: The aberrated image is filtered in the Fourier domain using an estimate of the neural transfer function (NTF) of the visual system. White Gaussian noise is added to simulate neural noise. Nestares et al.^12^ further refine this step by employing a set of Gabor filters modulated by the NTF. For each optotype, this approach generates a set of neural images, each corresponding to a Gabor filter tuned to a specific frequency. The outcome is a further degraded representation of the optotype, referred to as the “neural image”^13^.
3. Template Matching: This step simulates the recognition process in the human brain by comparing the neural image of a given optotype to a precomputed set of processed optotype templates. The comparison is conducted using a correlation-based metric. Nestares et al.^12^, and Watson and Ahumada^13^ employ a Bayesian approach, calculating the probability of each optotype in the alphabet matching the neural image of the optotype to be recognized. Fülep et al.^14^ instead use Pearson’s correlation coefficient to compare the neural image of the optotype with the noise-free neural image of each letter in the alphabet. In each case the letter with more probability^12,13^ or higher correlation coefficient^14^ is “recognized” by the algorithm. This step yields a set of correlation metrics used to determine the recognized optotype.
4. VA Determination: The final step of the functional models is determining the VA using methods similar to those employed in the clinical practice and based on the percentage of optotypes in a line that are recognized by the patient^12–14^.

Functional models are quite complex and computationally intensive. Moreover, they depend on parameters such as NTF or the amount of neural noise that vary between individuals and are quite difficult to estimate or measure. Therefore, functional models tend to use “average” values of NTF and variance of neural noise. However, as shown by Watson and Ahumada^13^, an average NTF should be modified to fit the experimental data of a given patient. This calibration of the parameters of the model is common for all three functional models. Therefore, it is difficult to extrapolate these models for a given individual or population without performing a previous per subject calibration. Finally, the effect of accommodation is not explicitly considered in these models.

In recent years, several studies have applied decision-tree–based machine learning models to predict visual acuity outcomes in clinical contexts, such as after anti-VEGF treatment^15^ or refractive surgery^16^. These models rely on demographic and clinical predictors—e.g., baseline visual acuity, age, or systemic parameters—without explicitly including optical descriptors of image formation such as Zernike coefficients or pupil size. In this sense, our approach is conceptually aligned with those studies, but differs in scope by focusing on predicting visual acuity in healthy subjects using optical descriptors directly related to image formation. Moreover, unlike previous studies that analyzed heterogeneous clinical datasets from patients with ocular pathologies, our work is based on healthy subjects examined under a single, standardized measurement protocol, ensuring high data consistency and quality.

In this work, we propose machine learning-based approaches to overcome the main drawbacks of phenomenological and functional models. The first method is a machine learning approach based on Regression Trees^17^ and this model is akin to the phenomenological ones. Our second method is a functional algorithm using deep learning in the template matching step. To train these algorithms, we have carried out a clinical trial with a set of 135 individuals (270 different eyes) considering ocular aberrations, age, and amplitude of accommodation (AA) as variables for our models. The results show that regression trees-based methods can predict the VA of a given population with good accuracy and processing time without requiring any per subject calibration. Moreover, the proposed deep learning approach also provides good accuracy in the template matching step, showing potential to replace traditional template matching algorithms for VA functional modelling with considerably low computational cost (once the network is trained).

## Methods

In this work, we propose three methods to automatically compute the VA of a subject. The predictions are based on measured eye Zernike coefficients and age, with some models also incorporating the amplitude of accommodation or pupil size. These variables are well known to strongly affect the eye’s optical image formation process, which in turn determines retinal image quality and ultimately VA. For example, Watson and Ahumada^13^, showed that optical aberrations, represented by Zernike coefficients, affect the eye’s ability to identify optotypes, directly impacting VA. Two proposed methods employ regression trees using tabulated data, while the other uses a neural network to make predictions based on a previous classification of aberrated optotypes.

### Data acquisition: clinical trial

In the clinical trial, we obtained different parameters from 135 subjects (270 eyes), consisting of the 36 first Zernike coefficients, measured under mesopic conditions and recalculated for a 3 mm pupil, amplitudes of accommodation, corrected and uncorrected VA values, subject age and defocus curves. These data were used to train and test the proposed VA prediction methods.

The investigation was carried out at the University Clinic of the Faculty of Optics and Optometry at the Complutense University of Madrid, over a 7-month period (January–July 2024). The study followed the center’s clinical protocols and guidelines, and it was reviewed and approved by the Ethics Committee of the Clínico San Carlos University Hospital of Madrid (approval number Ref: 23/039-E). All patients included in the study signed a written informed consent preoperatively, after being fully informed about the purpose of the study. The study followed the tenets of the Declaration of Helsinki.

Eligible participants were between 30 and 65 years old, had a monocular best-corrected visual acuity (BCVA) of at least 0.10 logMAR, and a refractive error ranging from + 4.00 to −7.00 diopters (D) (spherical equivalent). Subjects with prior ocular surgery or pathologies such as amblyopia or strabismus were excluded. The protocol included the measurement of visual acuity, amplitude of accommodation (AA) and defocus curve (DC). All tests were conducted monocularly. Visual acuity was measured at far distance without (uncorrected distance VA (UDVA)) and with the best correction (BCVA). Subjective refraction to obtain the BCVA was performed using the Maximum Plus Maximum Visual Acuity (MPMVA) technique with an ETDRS chart (Precision Vision, USA) positioned at a distance of 4 m under photopic conditions (85 cd/m^2^). To simulate optical infinity, a vergence correction was applied by adding − 1/d (in meters) to the spherical component after completing the subjective refraction, where d represents the distance from the patient’s corneal vertex to the optotype. In this study, a simplified correction of − 0.25 D was used, which corresponds to a 4 m testing distance. Additionally, any letters incorrectly identified by the patient were also documented.

Table S1 shows more detail on subject demographics (age distribution, refractive error range, and amplitude of accommodation). The sample size was calculated using the statistical software Granmo 6.0 (Institut Municipal d’Investigació Mèdica, Barcelona, Spain). As the reference variable, we used the results of amplitude of accommodation from the study by Leon et al.,^18^ accepting an alpha risk of 0.05 and a beta risk of 0.2 in a two-sided test, and considering a 20% dropout rate. Since no reference data were available for the last group, the same sample size as in the previous group was assumed.

AA was assessed using the push-up method. A near ETDRS chart (Precision Vision, USA) was slowly moved toward the participant until the first consistent blur was reported. This was repeated three times, and the mean of the three measurements was used to calculate the amplitude of accommodation in diopters using the formula AA = 100/*d*, where *d* represents the mean distance (in cm) between the eye and the chart at that blur point.

For the defocus curve, BCVA was recorded at a distance of 4 m, for the dominant eye at far distance, while sequentially introducing trial lenses in 0.50 D steps ranging from − 3.00 to + 1.00 D. To prevent the subject from memorizing the letters, the chart was changed for each eye measurement. Each measurement was recorded using logMAR notation during the evaluation of UDVA, BCVA, and the defocus curve.

To determine sensory dominance at far distances, a + 1.50 D lens was alternately placed in front of each eye while the subject fixated on an ETDRS chart positioned at 4 m. The eye in which the blur was more tolerable was classified as the non-dominant eye, and the other as the dominant eye. This process was repeated to ensure consistency.

The Visionix Vx110 (Visionix Luneau, Chartres, France) is a device that integrates objective refraction, corneal topography, and aberrometry in a single platform. It measures higher-order aberrations up to the seventh order of the Zernike polynomial series, using a Hartmann-Shack sensor with near-infrared light at a wavelength of 800 nm. Aberrometry was performed under mesopic conditions, and measurements corresponding to a standardized 3 mm pupil diameter were selected for analysis, and the aberrations were recalculated for a standardized 3 mm pupil diameter.

### Regression trees

Predicting VA from a set of clinical measurements, such as those described in the clinical study, can be considered a regression problem. Machine Learning (ML) algorithms are well suited for this kind of problems if we have enough data to train and verify the algorithms. In this context, we developed two methods to estimate VA based on regression trees that allow predicting continuous values from tabulated data by dividing the data into subsets using decision rules. Both methods use ensemble learning techniques, combining multiple regression trees to achieve robust and accurate predictions. Specifically, we applied gradient boosting, an iterative approach that combines several weak predictive models—each slightly better than random guessing—to construct a stronger overall predictive model. In gradient boosting, each subsequent regression tree is trained to reduce the residual errors from previously built trees by minimizing a predefined loss function^19^. The first method utilizes Least Squares Boosting (LSBoost), employing mean squared error as the loss function. In each iteration, LSBoost fits a regression tree to the residuals from previous iterations using gradient descent optimization^19^. The other approach employs Extreme Gradient Boosting (XGBoost) that extends the gradient boosting concept with several performance-enhancing optimizations. Among its most important improvements are regularization (L1 and L2) to prevent overfitting, efficient use of memory and computation time, and a histogram-based tree construction technique that speeds up processing for large data sets. In addition, it incorporates parallel processing^20^.

#### Data used for training and testing

Since regression tree-based methods are specifically designed for tabulated data, we used the Zernike coefficients measured under uncorrected conditions as a baseline. From these, we generated modified versions to represent corrected vision and defocus curve conditions by adjusting the defocus-related terms^21^. These coefficients, along with amplitude of accommodation, age, and VA, were used for training and testing both predictive models. We include in our model the role of accommodative compensation which acts only when the defocus is negative. Therefore, the amplitude of accommodation value corresponding to each subject is applied only when the defocus is negative; otherwise, it is set to zero. This simulates that the eye is not exerting any accommodative effort for positive defocuses.

Our dataset comprises 1744 observations derived from a total of 135 subjects. Each subject contributed data from both eyes, resulting in 270 baseline measurements under uncorrected conditions, in which Zernike coefficients were clinically measured. To simulate corrected vision, we generated an additional 270 observations by modifying the defocus-related Zernike terms. Furthermore, defocus curves were obtained for the dominant eye of each subject at 9 different defocus levels. These conditions were also simulated by altering the defocus component of the baseline Zernike coefficients, yielding 135 × 9 = 1215 additional observations. This resulted in an initial dataset of 270 (uncorrected) + 270 (corrected) + 1215 (defocus) = 1755 observations. After removing 11 entries due to missing VA data, the final dataset consisted of 1744 observations. These were randomly partitioned into a training set (80%) and a test set (20%) for model development and evaluation.

### Neural network

A neural network for image classification is a mathematical model that takes an image as input, processes the information through a sequence of layers to identify key features and finally predicts a label. In this case, the network is not trained on tabulated data (unlike regression trees), but instead it uses as input simulated aberrated optotypes generated from the tabulated data. Thus, this method aims to predict the visual acuity of subjects based on synthetic images that simulate their corresponding neural images derived from a set of optotypes. Rather than predicting VA directly, the network classifies each optotype image as either recognized or unrecognized. The overall VA is then inferred following a standard clinical protocol: for each VA level, a line of five aberrated optotype images is generated and evaluated by the network. If the network fails to recognize all images in a given line, the test is terminated. The final VA is computed by taking the VA value of the last attempted line and adding 0.02 LogMAR units for each misclassified optotype. Additional details on this procedure are provided in section “Determination of VA by the neural network”. Therefore, this method indirectly estimates VA by simulating the clinical process of visual recognition, using a neural network to classify optotypes across different sizes.

#### Generation of aberrated optotypes

The first step is to create a sharp, undistorted optotype image of the character to be evaluated using the Sloan font, ensuring that the optotype size is appropriate for the VA being evaluated. Next, this optotype image is blurred by convolution with the subject’s Point Spread Function (PSF). The PSF is generated from the complex pupil function that considers the subject’s aberrations defined with the Zernike coefficients, the Styles-Crawford effect, the wavelength and the pupil radius. Then, a retinal sampling process is performed to model image acquisition by the subject’s retina^12^. The final step is considering the neural processing of the retinal information by means of the Neural Transfer Function (NTF). This function is defined as the quotient between the Contrast Sensitivity Function (CSF) and the Optical Transfer Function (OTF)^13^. The OTF is a mathematical representation that describes how an optical system modulates the spatial characteristics of an image, both in terms of amplitude and phase, while the CSF is a representation of the visual system’s ability to detect contrast stimuli at different spatial frequencies.

The individual NTF can be measured by aberration-free projection systems or can be computed from the measured CSF^22^. In this work we have used the average NTF obtained from the standard CSF (SCSF) proposed by Watson and Ahumada^23^, and the Mean Optical Transfer Function (MOTF) from the same authors^13^. This approach provided excellent results in their visual acuity model. The SCSF is given by Eq. (1)

1 $$SCSF\left(f\right)={\Psi\:}\:\text{sech}\left[{\left(\frac{f}{\varphi\:{f}_{0}}\right)}^{p}\right]\:-a\:\text{sech}\left[\frac{f}{\varphi\:{f}_{1}}\right],$$

where $\:f$ represents frequency in cycles/degree, $\:{\Psi\:}\:$is a gain factor, $\:{f}_{0}$ and $\:{f}_{1}$ represent scale frequencies in the high-frequency and low-frequency lobes respectively, *a* determines the weight of the low-frequency lobe, *p* is a fitting coefficient and $\:\varphi\:$ represents the frequency scale. The values of these parameters were obtained from^23^ and are shown in Table 1.

**Table 1:** Parameters used for the computation of the SCFS in Eq. (1) as introduced in^23^. The frequencies $\:{f}_{0}$ and $\:{f}_{1}$ are given in cycles/degree.

$\:{\Psi\:}$	$\:{f}_{0}$	$\:{f}_{1}$	a	*p*	$\:\varphi\:$
373.08	4.1726	1.3625	0.8493	0.7786	1

**Fig. 1:**
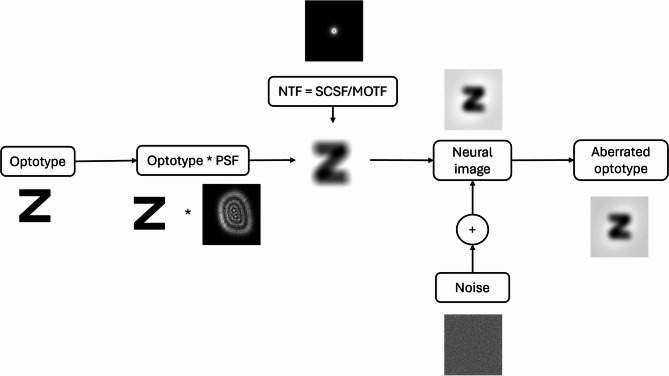
Processing pipeline to compute simulated and aberrated optotypes to be used for the training and evaluation of the recognition neural network.

The model for the MOTF, as presented in^24^, is given by Eq. (2)

2 $$\:MOTF\left(f\right)=0.426\times\:\frac{{e}^{-0.028\:f}}{1+\frac{AF}{7}}+0.574\times\:{e}^{-0.37\:f}+0.123\times\:\frac{{e}^{-37\:f}}{1+\frac{AF}{7}}+0.877\times\:{e}^{-360\:f},$$

where the frequency *f* is given in cycles/degree, and $\:AF$ is an age factor defined by $\:AF=1+{\left(age/D\right)}^{4}$, *D* being a normalization parameter with a value of 70 years. The NTF is finally obtained as SCSF/MOTF. The last step of the process to generate the aberrated optotype is to add a random Gaussian noise with zero mean and a variance of 0.01. A summary of the different steps is given in Fig. 1. This sequence of steps aims to simulate not only the optical aberrations but also the retinal and neural processing, to closely reproduce the subject’s actual visual perception.

#### Neural network architecture

Our neural network is designed for image classification and consists of several key processing stages. The network begins with an image input layer of size 224 × 224 pixels, followed by a feature extraction block comprising a 3 × 3 convolutional layer with 8 filters, batch normalization, and a ReLU activation function to introduce nonlinearity. To reduce spatial dimensions while retaining essential features, a max-pooling layer with a 2 × 2 filter and stride of 2 is applied. The classification stage consists of a fully connected layer with a number of neurons equal to the number of target classes (i.e., 2 in this case). This layer is followed by a softmax activation function, which converts the activations into class probabilities. Finally, a classification layer computes the categorical cross-entropy loss for model optimization.

#### Training and data used

The model was trained using Adam optimizer, with batches of 32 images over 10 epochs. We used a total of 14,700 images which were divided into 70% for training, 15% for validation and 15% for testing. Each image was labeled based on the subject’s response during the clinical trial. Specifically, if the subject correctly identified the letter shown in the image, it was labeled as “recognized”; otherwise, it was labeled as “not recognized.” This binary labeling derived from the subject’s answers served as the ground truth for training the network.

In this case, the grouping differs from that used in regression trees because for this case, we only included the Zernike coefficients of uncorrected subjects (in addition to age and uncorrected VA) as they enable a more accurate simulation of what the subject sees. For example, in the case of negative defocus, the amplitude of accommodation would have to be taken into account to determine the appropriate defocus value. However, there is no precise information on the actual accommodation of each subject. Considering these factors, we utilized Zernike data from 210 eyes across 14 visual acuity levels (ranging from 1 logMAR to −0.3 logMAR, in steps of 0.1 logMAR), with five letters per visual acuity level, resulting in a total of 210 × 14 × 5 = 14,700 images.

#### Determination of VA by the neural network

To predict a subject’s VA using the neural network, we follow the same procedure employed in clinical practice. The network is provided with five aberrated optotypes of a specific VA value, with VA ranging from 1 logMAR to −0.3 logMAR in steps of 0.1. The procedure begins with five aberrated optotypes at a VA of 1 logMAR. If the network classifies at least one of the five optotype as recognized, the VA is decreased by 0.1 (VA to 0.9 logMAR), and the process continues in this way until the network classifies all five optotypes in a line as not recognized. At this point, the process stops, and the VA is calculated by multiplying the total number of errors by 0.02 logMAR units and then adding the result to the VA value of the last line attempted. An example of this VA calculation is shown in Figure S1.

## Results

Next, we use the proposed approaches to determine the VA from a set of Zernike coefficients, age and in some cases individual measurements of amplitude of accommodation to account for subject accommodation variability (regression trees) and pupil size (neural network). Table 2 presents a comparison of the variables used in each model. We show that our proposed approaches can provide fast and near-clinical accuracy VA predictions from experimental data obtained from the clinical trial.

**Table 2:** Comparison of variables used for training and prediction with regression trees and generate optotypes for the neural network.

	Regression trees	Neural network
Zernike coefficients		
Uncorrected (measured)	Yes	Yes
Corrected	Yes	No
Defocus curves	Yes	No
Age	Yes	Yes
Amplitude of accommodation	Yes	No
Pupil size	No	Yes
Visual acuity	Yes	Yes

### Regression trees

As the clinical trial measured 36 Zernike polynomials for each subject, we evaluated which subset of Zernike coefficients yielded the best results for both methods (LSBoost and XGBoost). Several tests were conducted to determine the optimal number of coefficients evaluated over the test set aiming to achieve the best performance. Specifically, we assessed the following subsets of Zernike coefficients: 5 (from the 1st to the 5th coefficients in the OSA convention), 9 (from the 1st to the 9th), 14 (from the 1st to the 14th), and 36 (from the 0th to the 35th). To perform these tests, we used as input the Zernike coefficients, age and corresponding VA of the test set. In Table 3, we show the results obtained for the LSBoost approach. In this table, we provide usual metrics such as the mean square error (MSE), the root mean square error (RMSE), the mean absolute error (MAE), the R^2^ metric representing the goodness of the fitting, the maximum and minimum errors (MaxE and MinE) and the mode and median of the errors. For context, an RMSE of 0.1 logMAR approximately corresponds to one line difference on a standard visual acuity chart. Therefore, RMSE values below 0.1 indicate good predictive performance. The best result for each metric is highlighted in bold. As shown in Table 3, the results obtained using 9 and 14 Zernike coefficients are very similar, with the 9 Zernike coefficients providing the best overall performance. A visual representation of the MSE, RMSE, and MAE results are presented in Figure S2(a).

**Table 3:** Metrics obtained with LSBoost approach and different number of Zernike coefficients in the test set. All metrics are expressed in logMAR, except for MSE, which is in logMAR^2^, and R^2^, which is dimensionless.

# Zernike coefficients	5	9	14	36
MSE	0.0075	**0.0063**	0.0065	0.0071
RMSE	0.0868	**0.0792**	0.0809	0.0840
MAE	0.0601	**0.0574**	0.0575	0.0585
R^2^	0.8937	**0.9115**	0.9077	0.9004
MaxE	0.4800	**0.4200**	**0.4200**	0.4600
MinE	**0.0000**	**0.0000**	**0.0000**	**0.0000**
Mode	**0.0000**	**0.0000**	**0.0000**	**0.0000**
Median	**0.0400**	**0.0400**	**0.0400**	**0.0400**

Best values are in bold.

Therefore, we used 9 Zernike coefficients as input in our LSBoost approach for subsequent experiments. We then incorporated the measured amplitude of accommodation of each subject as an additional parameter observing improvements in most metrics (MSE, RMSE, MAE, R^2^, etc.) except for the mode. These results are shown in Table 4(a). Subsequently, in Table 4(b), we provide computed results when outliers in the test set were removed (i.e., errors greater than 3σ). Note that the data used to train and evaluate these models were obtained from a clinical trial. Therefore, we must assume the possibility of erroneous entries, whether from false-positive or false-negative responses by participants, or mistakes made by participants and/or clinical staff. The number of entries evaluated in Table 4(b) are 348 and 328 with and without removing outliers respectively. Consequently, the percentage of removed outliers in the test set is 5.75%. To assess the model’s generalization ability, Table 4(c) presents the results obtained for the training set, including the amplitude of accommodation. Comparing these results with those from the test set (Table 4(a) and (b)) provides insight into potential overfitting or underfitting. As expected, the training set exhibited lower error values across all metrics. However, the model maintained strong generalization, with a slight increase in error on the test set but no indications of severe overfitting. These findings confirm that the model performs well on unseen data while preserving robustness. A visual representation of the percentage improvement derived from these metrics is provided in Figure S2(b). Finally, Figure S3 shows plots of the clinically measured VA (real VA) versus the LSBoost-predicted VA (predicted VA) for the test set, presented both with outliers (left) and without outliers (right). The color of each point reflects the absolute error between the real and predicted VA.

**Table 4:** Comparison of metrics for LSBoost approach with and without amplitude of accommodation (a) and with and without considering outliers including amplitude of accommodation (b), both calculated using the test set; (c) shows the results computed using the training set including amplitude of accommodation. All metrics are expressed in LogMAR units, except for MSE, which is in LogMAR^2^, and R^2^, which is dimensionless.

	MSE	RMSE	MAE	*R* ^2^	MaxE	MinE	Mode	Median
(a)								
Without AA	0.0063	0.0792	0.0574	0.9115	0.4200	0.0000	**0.0000**	0.0400
With AA	**0.0058**	**0.0760**	**0.0559**	**0.9184**	**0.3400**	**0.0000**	0.0600	**0.0400**
(b)								
With outliers	0.0058	0.0760	0.0559	0.9184	0.3400	**0.0000**	**0.0600**	**0.0400**
Without outliers	**0.0033**	**0.0576**	**0.0465**	**0.9485**	**0.1400**	**0.0000**	**0.0600**	**0.0400**
(c)								
With outliers	0.0007	0.0267	0.0179	0.9901	0.1200	**0.0000**	**0.0000**	**0.0200**
Without outliers	**0.0004**	**0.0199**	**0.0138**	**0.9946**	**0.0400**	**0.0000**	**0.0000**	**0.0200**

Best values are in bold.

The same tests were conducted using the XGBoost approach. Table 5 shows the results obtained with different numbers of Zernike coefficients. As shown in Table 5, while the XGBoost approach yields similar results across the various cases, the results obtained using 5 Zernike coefficients are slightly better than those from the other configurations. A visual representation of the MSE, RMSE, and MAE results are presented in Fig. S2(c).

**Table 5:** Metrics obtained with XGBoost approach and different number of Zernike coefficients. In the last column, it is shown the metrics with amplitude of accommodation and using the 9 Zernike coefficients. All metrics are expressed in logMAR, except for MSE, which is in logMAR^2^, and R^2^, which is dimensionless.

# Zernike coefficients	5	9	14	36	9AA
MSE	**0.0070**	**0.0070**	0.0081	0.0071	0.0075
RMSE	**0.0836**	**0.0836**	0.0902	0.0842	0.0866
MAE	**0.0580**	0.061	0.0658	0.0590	0.0628
R^2^	**0.9014**	0.9013	0.8852	0.9000	0.8943
MaxE	0.4000	0.4000	0.4800	0.4800	**0.3600**
MinE	**0.0000**	**0.0000**	**0.0000**	**0.0000**	**0.0000**
Mode	**0.0200**	**0.0200**	**0.0200**	**0.0200**	**0.0200**
Median	**0.0400**	**0.0400**	0.0600	**0.0400**	0.0500

Best values are in bold.

Consequently, we proceeded to remove the outliers in the test set, following the same procedure as with the LSBoost approach. The corresponding results are presented in Table 6(a) and Figure S4. In this case, the number of removed outliers is 16, representing 4.6% of the test set. To evaluate the model’s ability to generalize, Table 6(b) shows the results obtained for the training set. The model demonstrates strong generalization capabilities, as evidenced by lower error metrics in the training set and a reasonable increase in error in the test set. While the test set exhibits a higher maximum error, particularly before outlier removal, the overall error distribution remains consistent. The minimum error remains stable across all cases, and slight differences in mode and median values further indicate effective generalization without significant overfitting. A visual representation of the percentage improvement derived from these metrics is provided in Figure S2(d).

**Table 6:** Comparison of metrics for XGBoost approach with and without outliers when using the 5 Zernike coefficients and the test set (a) and the training set (b).

	MSE	RMSE	MAE	*R* ^2^	MaxE	MinE	Mode	Median
(a)								
With outliers	0.0070	0.0836	0.0580	0.9014	0.4000	**0.0000**	**0.0200**	**0.0400**
Without outliers	**0.0040**	**0.0629**	**0.0484**	**0.9386**	**0.1800**	**0.0000**	**0.0200**	**0.0400**
(b)								
With outliers	0.0006	0.0241	0.0156	0.9919	0.1200	**0.0000**	**0.0000**	**0.0200**
Without outliers	**0.0004**	**0.0191**	**0.0129**	**0.9950**	**0.0400**	**0.0000**	**0.0000**	**0.0200**

Best values are in bold.

Finally, comparing Tables 4(b) and 6(a), we see that LSBoost outperforms XGBoost across most error metrics, demonstrating lower overall prediction errors and a better fit to the data. It also shows greater robustness to extreme values, with a consistently lower maximum error. After outlier removal, both models improve in all performance metrics, with LSBoost showing slightly better results. Although the differences between the two models become less pronounced without outliers, XGBoost appears more sensitive to extreme values. Overall, LSBoost exhibits superior predictive accuracy and generalization. Section “Computation time” discusses the computation time required for training and prediction, and section “Comparison with other models” presents a comparison of the LSBoost and XGBoost models with other approaches.

Age was included as a parameter in our models due to its well-established influence on VA, which tends to decrease with increasing age. Age-related changes in the visual system can significantly affect daily activities and quality of life, with common alterations including reduced VA^25^.

Radner and Benesch examined the age-related progression of best-corrected VA in healthy eyes between the ages of 25 and 74^26^. Their findings indicated that VA remained consistently high up to 54 years of age, with a noticeable decline beginning between 55 and 59 years. In healthy eyes up to age 64, a minimum angle of resolution of less than 1 min of arc (corresponding to a visual acuity better than 0.0 logMAR) could still be expected. In contrast, Frisén et al.,^27^ reported that peak visual acuity was reached at approximately 25 years of age.

To illustrate the contribution of age to VA, we analyzed models using 9 Zernike coefficients combined with amplitude of accommodation for LSBoost, and 5 Zernike coefficients for XGBoost—the configurations that yielded the best results for each model. When age was removed from the models, performance declined notably. In LSBoost model, MSE increased by 30% (0.0063 to 0.0082), RMSE by 14% (0.0792 to 0.0906), and MAE by 13% (0.0574 to 0.0651), while R^2^ decreased by about 3% (0.9115 to 0.8842). Similarly, in the XGBoost model, MSE increased by 19% (0.0070 to 0.0083), RMSE by 9% (0.0836 to 0.0910), and MAE by 13% (0.0580 to 0.0655), while R^2^ decreased by about 2% (0.9014 to 0.8832). These results highlight the significant contribution of age in improving the accuracy of VA prediction. Moreover, this contribution can be observed in Figure S5, which shows the feature importance for each model. Age appears as the second most important feature in LSBoost and as one of the top-ranked features in XGBoost (several features share the maximum importance score). The bar plots also reveal that $\:{Z}_{2}^{0}$ is the most influential feature in LSBoost, and it holds a similar level of importance to age in XGBoost.

Finally, we performed two stratified analyses: age and refractive error. In the case of age, the data were divided into six groups: 30–40, 41–44, 45–50, 51–55, 56–60, and 61–65 years. Comparing the results for groups without outliers for both LSBoost and XGBoost to the overall test set, we observe that the 30–40 and 41–44 groups outperform the test set, the 61–65 group performs similarly to the test set, and the remaining groups perform worse. For LSBoost, the best metrics are observed in the 30–40 group (RMSE = 0.0411 vs. 0.0576 in the test set) and the worst in the 51–55 group (RMSE = 0.0716). For XGBoost, the best metrics are also in the 30–40 group (RMSE = 0.0507 vs. 0.0629) and the worst in the 45–50 group (RMSE = 0.086). Overall, model performance is better in younger groups (30–44), likely reflecting lower inter-subject variability in VA, whereas performance is worse in middle-age groups (45–55), where natural variability in VA is higher. The similarity between the 61–65 group and the overall test set likely reflects that VA in older adults tends to converge toward a common pattern of age-related changes, and the model predictions for this group approximate the test set average. The results obtained in this stratified analysis can be found in Table S2 for LSBoost and Table S3 for XGboost.

In the case of refractive error, we stratified the data using the spherical equivalent (SE), calculated as (3), based on the sphere (S) and cylinder (C) measurements obtained in the clinical trial.

3 $$\:SE=S+C/2,$$

Groups were defined as myopic (SE < − 0.5 D), emmetropic (− 0.5 D ≤ SE ≤ 0.5 D), and hyperopic (SE > 0.5 D). In this stratified analysis, we considered only 53 observations, fewer than the 348 in the full test set. As VA measurements for each subject were obtained in both eyes under several optical conditions: uncorrected, corrected (second-order Zernike terms set to zero), and defocus curves (${Z}_{2}^{-2}$ and $\:{Z}_{2}^{-2}$ set to zero and varying $\:{Z}_{2}^{0}$ according to the defocus level), in test set there are observations under all of these optical conditions. However, for the refractive error analysis, we used only the original Zernike measurements representing the actual optical state of each eye. This ensures that the analysis reflects model performance across real refractive categories. When examining the results, both myopic and hyperopic groups perform worse than the overall test set for both LSBoost and XGBoost (myopic: RMSE = 0.1177 for LSBoost, 0.0949 for XGBoost; hyperopic: RMSE = 0.1522 for LSBoost, 0.1434 for XGBoost, compared to RMSE = 0.0576 and 0.0629 for the full test set, respectively). In contrast, the emmetropic group outperforms the test set in both models (RMSE = 0.0480 for LSBoost and 0.0498 for XGBoost). All RMSE values reported here refer to metrics calculated without outliers. For more metrics without outliers and metrics with outliers see Table S4.

### Neural network

As previously explained, the neural network attempts to recognize each neural optotype image by classifying it into a recognition label (0 or 1, depending on whether the network recognizes the optotype image). This classification is then used, as detailed in section “Determination of VA by the neural network”, to calculate the VA. Therefore, the effectiveness of this approach in estimating VA is directly linked to the neural network’s ability to recognize the neural optotypes. To assess the robustness of the trained neural network, we computed the confusion matrix on the test set, which is presented in Table 7. As shown in Table 7, the network correctly identifies 93.2% of the optotypes recognized by the subjects (true positives) and 85.3% of the optotypes not recognized by the subjects (true negatives), thus the sensitivity of the neural network is 93.2% with a specificity of 85.3%. However, the network exhibits a 14.7% false positive rate and a 6.8% false negative rate.

**Table 7:** Neural network performance on the test set. (a) confusion matrix: the horizontal axis represents the actual classes (target), while the vertical axis shows the predicted classes by the network. The black values in the cells indicate the number of optotypes classified by the network for each class. Bold and italic percentages indicate correctly and incorrectly classified optotypes, respectively. (b) performance metrics derived from the confusion matrix, including sensitivity (recall), specificity, precision for each class, and overall accuracy.

(a) Confusion matrix				
**Output class**	**Unrecognized**	870	150	**85.3%**
				*14.7%*
	**Recognized**	81	1104	**93.2%**
				*6.8%*
		**91.5%**	**88%**	**89.5%**
		*8.5%*	*12%*	*10.5%*
		**Unrecognized**	**Recognized**	
		**Target class**		

	(b) Performance metrics			
	Sensitivity (Recall)	Specificity	Precision	Accuracy
**Unrecognized**	85.3%	88%	91.5%	89.5%
**Recognized**	93.2%	91.5%	88%	

To evaluate the network’s ability to predict VA, we used images generated from Zernike coefficients of subjects who were not included in the training and validation datasets, as the responses (i.e., the letters identified) from their VA measurement tests were unavailable. However, the VA values themselves were available. This set was composed of 27 individuals generating 54 cases. After making the predictions and performing the corresponding calculations, the network achieved a root mean square error (RMSE) of 0.2095 and a mean absolute error (MAE) of 0.1715. Section “Computation time” discusses the computation time required for prediction. These computed errors are significatively higher than the ones computed with the regression trees showing lower robustness of the neural network method to estimate visual acuity.

### Computation time

In our study, we emphasize computational speed because the aim of our models is the design and manufacture of customized ophthalmic lenses. In the patented method for ophthalmic lens design (US11092823B2)^28^, the subject’s VA profile is used to generate lens parameters. These parameters are optimized iteratively using VA based merit functions. Because the generation of lens parameters directly depends on predicted VA and involves repetitive optimization, the predictive model must provide results rapidly to avoid computational bottlenecks. This requirement justifies the use of fast algorithms, which enable the efficient integration of VA prediction into the design-to-manufacture process.

Among the three methods proposed, XGBoost approach is the fastest, followed by the LSBoost method. In terms of training time, XGBoost takes 3 min and 31 s, while LSBoost requires approximately 2 min. After the training phase, when predicting a single VA value from a set of Zernike coefficients, age and amplitude of accommodation, XGBoost takes approximately 1 ms, whereas LSBoost requires 116.243 ms. On the other hand, the estimation of VA by the neural network requires a more complex pipeline which impacts the computation time. Moreover, the computation time depends on the actual VA value itself: the lower the VA logMAR value, the longer it takes, since it must evaluate more neural optotypes. As example, a VA of − 0.18 logMAR requires 23.558 s, while a VA of 0.62 logMAR requires 14.868 s. When predicting all 348 observations in the test set, XGBoost requires only 1 ms, regression trees with LSBoost take approximately 145.5 ms, and the neural network requires around 3 h. In this case, when we refer to “the neural network,” we mean the entire processing pipeline, including the simulation of multiple optotypes at different VA levels and the corresponding classification by the neural network. The network itself requires only ~ 2.5 ms to classify a single optotype. When optotype generation is included, the time increases to ~ 0.44 s per optotype. Since each observation may involve up to 15 VA levels (form 1 logMAR to −0.3 logMAR in steps of 0.1) with 5 optotypes per level, the maximum time per observation (corresponding to the prediction of a single VA value) is ~ 33 s. For the 348 observations in the test set, this corresponds to a maximum total processing time of ~ 3.2 h. This time represents an upper bound, as the procedure often stops earlier once a given VA level cannot be recognized (i.e., when all five optotypes of that level are classified as “not recognized”), thereby reducing the actual runtime. These processing times have been obtained by always the same computer. The experiments were conducted on a system equipped with an Intel Core i9-11900 CPU, 128GB of RAM, a NVIDIA RTX A5000 GPU, and a 1 TB SSD. The software environment includes MATLAB (version R2023a), which was used for implementing LSBoost and the neural network models. For XGBoost, we used a Python 3.10.17 environment, including key dependencies such as NumPy 2.2.5, SciPy 1.15.2, scikit-learn 1.6.1, and XGBoost 3.0.0. This Python environment was called directly from MATLAB. The system runs on Windows 11.

### Comparison with other models

Regarding the functional VA models, Watson and Ahumada^13^ reported a RMSE of 0.056 logMAR, while the RMSE for LSBoost was 0.058 logMAR and 0.063 logMAR for XGBoost, in both cases after removing outliers. Therefore, the results are comparable between our model and that of Watson and Ahumada, though XGBoost shows a slightly higher RMSE. However, there are significant differences in the size of the test set. Watson and Ahumada used a set of 4 subjects and obtained 152 observations using a defocus curve, whereas we verified our algorithms on a larger test set with a total of 348 observations coming from a total of 20 subjects. Thus, our results are supported by greater statistical power. Additionally, the model proposed by Watson and Ahumada employs multiple possible values for a parameter ($\varphi$) that cannot be directly measured in patients and is adjusted to optimize the fitting. In contrast, our LSBoost and XGBoost based methods do not use any phenomenological parameter and the neural network employs a single common $\varphi$ value across all subjects. The model of Nestares et al.^12^ was verified by Dalimier et al.^21^, who used 10 subjects with 140 observations and obtained a RMS of 0.23 in decimal visual acuity, which corresponds to 0.1 logMAR. Finally, Fülep et al.^14^ tested their model with a set of 8 subjects and reported an RMSE error of 0.045 logMAR. A comparative summary of the different functional VA models, including RMSE values and test set size, is provided in the Supplementary Material (Table S5). Therefore, we can conclude that our model and the functional VA models present good accuracy, with an average value close to 0.06 logMAR. This is interesting because, in the ETDRS chart commonly used to assess VA, the penalty for failing to recognize a letter corresponds to 0.02 logMAR. Thus, we can estimate the error of our models as equivalent to failing to recognize two letters on the ETDRS chart. Moreover, the regression tree-based models (LSBoost and XGBoost) present a significant computational advantage compared to all these models previously proposed, as they do not require image formation.

## Discussion

In this paper, we have presented three methods for estimating a subject’s visual acuity based on measured Zernike coefficients, age, and, in some cases, amplitude of accommodation. When comparing the VA results obtained from the regression tree models (LSBoost and XGBoost) to those from the classification network, the former demonstrates better performance. Compared to functional models of VA, we have obtained for both LSBoost and XGBoost a similar accuracy, with a RMSE close to 0.06 logMAR units, but the machine learning algorithm proposed are much faster and simple than any of the functional models.

Regarding the classification network’s performance, it achieves high accuracy in distinguishing between recognized (93.2%) and unrecognized (85.3%) optotypes, with an overall classification accuracy of 89.5%. Although this percentage is relatively high, it is not sufficient to achieve accurate VA estimates due to the calculation method: every five misclassifications by the neural network result in a 0.1 logMAR increase in the VA value.

Among the two regression tree approaches, LSBoost produces the best results when using nine Zernike coefficients and amplitude of accommodation. However, in terms of computational efficiency, XGBoost is the fastest method. Notably, XGBoost maintains a relatively stable computation time even as more cases are introduced, unlike LSBoost. Although both methods share the same theoretical basis of Gradient Boosting, they differ in their implementations and capabilities. LSBoost is a simpler, more classical variant, while XGBoost is designed to maximize efficiency and performance in more demanding contexts. Given that the VA results obtained with XGBoost are only slightly inferior to those of LSBoost, the choice between the two depends on whether higher accuracy or lower computational time is prioritized.

Since the input data consists of tabulated values, the regression tree models yield more consistent results than the neural network. This is expected, as neural networks are primarily designed for image-based tasks, while tabular data is better suited for machine learning approaches like regression trees. Achieving accurate VA predictions with neural networks is more challenging because it requires generating synthetic images that accurately simulate a subject’s perception. This process is complex, as each individual has unique CSF and MTF. To create a generalized model, a generic CSF and MTF were used, making the simulation less representative of actual visual perception. Moreover, the neural network indirectly estimates VA by classifying optotypes rather than predicting VA directly, following the procedure explained in section Determination of VA by the neural network. As a result, individual misclassifications propagate through the VA calculation, introducing variability in the estimated VA.

In contrast, boosting models such as LSBoost and XGBoost remain superior in this context for several reasons. First, they are naturally suited for tabular data and can exploit the structure of measured features directly. Second, the iterative combination of weak learners decreases both bias and variance, producing robust predictions even with relatively small datasets. Third, these models are not dependent on assumptions about visual perception or synthetic image generation, avoiding potential sources of error present in the neural network approach. Finally, boosting techniques present an advantageous compromise between precision and efficiency in computation, with LSBoost providing the highest precision and XGBoost achieving faster computation for larger datasets. Taken together, these elements explain why regression tree models outperform the neural network in predicting VA from tabulated measurements.

This study presents a model designed to replicate the clinical approach to visual acuity assessment, where optotype images are generated as perceived by the subjects, and a neural network acts as a recognizer. Additionally, we propose a model that achieves strong performance (LSBoost) and another that, while slightly less accurate, remains effective and offers faster computation (XGBoost).

In the case of neural network approach, its current implementation requires longer processing times due to the required complete pipeline consisting of the simulation of multiple optotypes at different VA levels and the corresponding classification by the neural network. In future work, one possible approach to accelerate this process would be to train a network to directly predict VA from the aberrated optotype; however, this would require a significantly larger dataset and a reliable simulation of the subject’s perception of the optotype, which is challenging to achieve accurately. Alternatively, a convolutional neural network could be designed to predict the aberrated optotype directly from the Zernike coefficients, potentially incorporating aspects of the neural processing of visual information. Such an approach could considerably reduce computation time by avoiding the repeated optotype simulation step.

In the case of regression tree models, it will be important to explore robust strategies for handling outliers. In this study, we excluded test set outliers exceeding 3σ, assuming that these outliers resulted from occasional errors in recording VA. Thus, results were reported both with and without these extreme values. However, we did not include any outlier handling strategy for the training or validation sets. Possible strategies could include robust loss functions (e.g., Huber or Tukey’s biweight^29^ during training, or weighting schemes that reduce the influence of extreme observations.

## Supplementary Information

Below is the link to the electronic supplementary material.

Supplementary Material 1

## Data Availability

The code and data are available at [https://github.com/aguesie/ML-VA-Prediction](https:/github.com/aguesie/ML-VA-Prediction).
