# Supplementary material for: Fast and accurate visual acuity prediction based on optical aberrations and machine learning
Source: Sci Rep. 2025 Dec 19;15:44177. doi: 10.1038/s41598-025-27972-0 (PMC12717265; doi:10.1038/s41598-025-27972-0)
Supplement: Supplementary file 1 — Supplementary Material 1 [file 41598_2025_27972_MOESM1_ESM.pdf]

Supplementary material

Fast and accurate visual acuity prediction based on optical aberrations and machine learning

A. Sierra[1*], I. Baoud Ould Haddi[2], S. Fernández-Núñez[3], J.A. Gómez-Pedrero[3], M. García-Montero[2], N. Garzón[2], J. Alonso[3,4], E. Pascual[4], J. Vargas[1*]

[1]Departamento de Óptica, Facultad de Ciencias Físicas
Universidad Complutense de Madrid, Plaza de Ciencias 1, 28040 Madrid, Spain
[2]Departamento de Optometría y Visión, Facultad de Óptica y Optometría
Universidad Complutense de Madrid, C. de Arcos de Jalón, 118 28037 Madrid, Spain
[3]Departamento de Óptica, Facultad de Óptica y Optometría
Universidad Complutense de Madrid, C. de Arcos de Jalón, 118 28037 Madrid, Spain
[4]Clinical Research Department, Indizen Optical Technologies, 28002 Madrid, Spain
* Correspondence: aguesier@ucm.es; jvargas@ucm.es

1. Example of visual acuity calculation

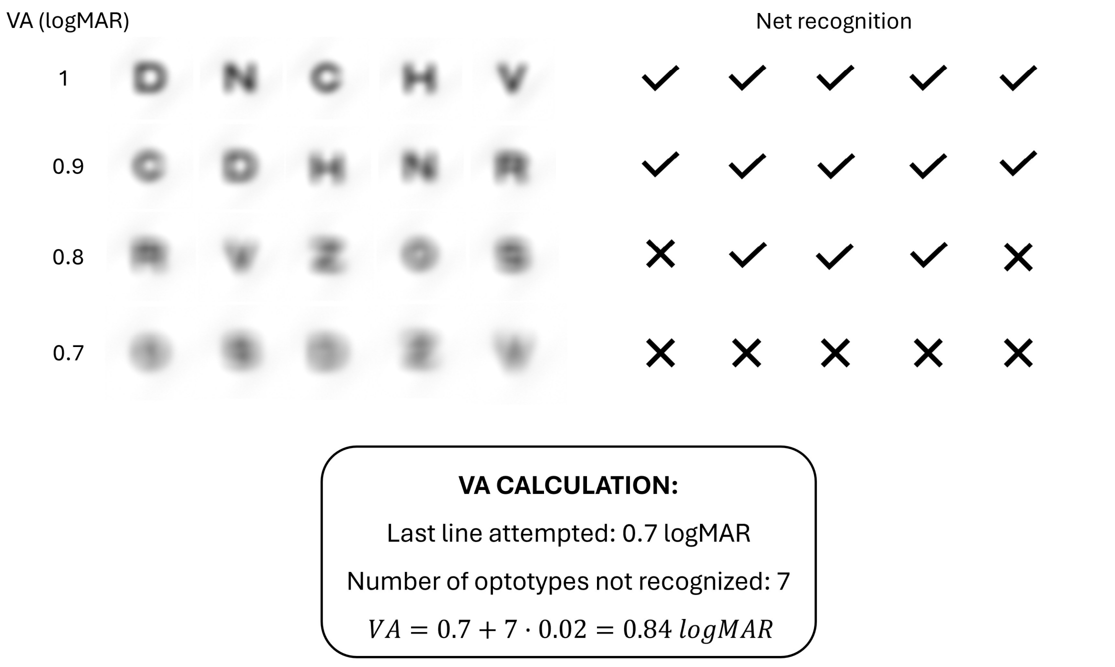

Figure S1. Example of the process used to calculate the VA using the neural network classification.

2. Clinical trial

Age group	Sample size	Age (years) (mean ± SD) (range)	Gender (male/female; %)	Spherical equivalent (D) (mean ± SD) (range)	Amplitude of accommodation (D) (mean ± SD) (range)
**Group 30-40**	66	34.5±3.3 (30.0 – 40.0)	36.4/63.6	-1.4±1.9 (-5.6 – 1.0)	6.7±1.8 (4.0 – 10.0)
**Group 41-44**	50	43.3±1.2 (41.0 – 44.0)	56.0/44.0	-0.6±1.4 (-5.1 – 3.5)	4.19±0.75 (3.23 – 5.00)
**Group 45-50**	34	47.9±1.7 (45.0 – 50.0)	47.1/53.0	-1.0±2.2 (-5.3 – 5.1)	2.38±0.55 (1.54 – 3.45)
**Group 51-55**	48	53.2±1.5 (51.0 – 55.0)	45.8/54.2	0.1±1.5 (-3.3 – 4.0)	2.42±0.79 (1.61 – 4.25)
**Group 56-60**	36	57.9±1.4 (56.0 – 60.0)	38.9/61.1	0.0±1.7 (-4.0 – 2.5)	1.49±0.48 (0.56 – 2.50)
**Group 61-65**	36	62.7±1.3 (61.0 – 65.0)	38.9/61.1	-1.0±2.9 (-6.9 – 2.4)	1.5±1.1 (1.1 – 3.2)

Table S1. Preoperative demographic data for the patients evaluated. SD means standard deviation and D, diopters. These patients were selected because accommodation is directly affected by the patient’s age. Therefore, six study groups were established according to subjects’ age, allowing for a more precise analysis.

3. Visual acuity prediction LSBoost and XGBoost

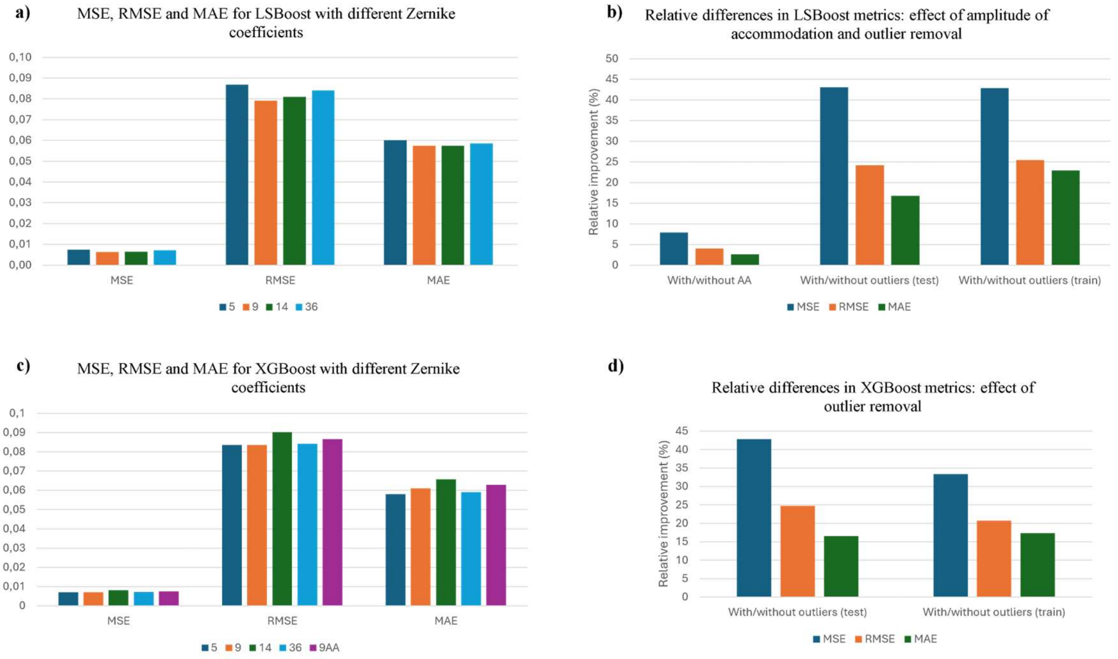

Figure S2. Bar charts comparison of LSBoost and XGBoost performance metrics under different conditions. (a) MSE, RMSE, and MAE computed by the LSBoost model for the test set using 5, 9, 14, and 36 Zernike coefficients. Metrics are expressed in logMAR units, except for MSE, which is in logMAR². Values correspond to those reported in Table 3. (b) Relative improvement (%) of LSBoost metrics (MSE, RMSE, MAE) under different conditions (with/without amplitude of accommodation and with/without outlier removal in training and test sets). Values derived from Table 4. (c) MSE, RMSE, and MAE for the XGBoost model for the test set using 5, 9, 14, 36 Zernike coefficients, and 9 Zernike coefficients with amplitude of accommodation. Metrics are expressed in logMAR units, except for MSE, which is in logMAR². Values correspond to those reported in Table 5. (d) Relative improvement (%) of XGBoost metrics (MSE, RMSE, MAE) with and without outlier removal in training and test sets. Values derived from Table 6.

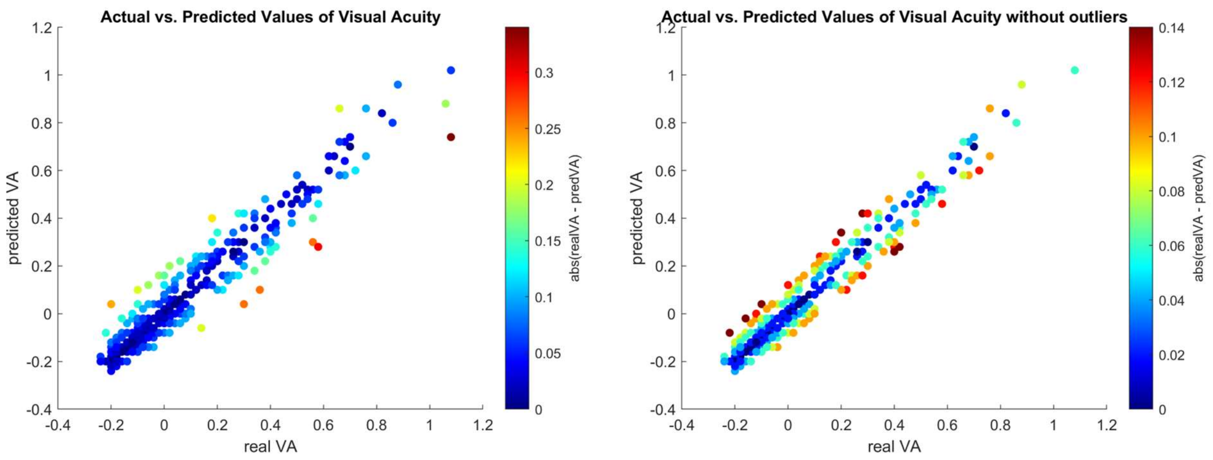

Figure S3. Plot showing the VA (in logMAR) as measured in the clinical trial (real VA) versus the LSBoost predicted VA (predicted VA) for the test set with (left) and without outliers (right). The color of the points is given by the absolute error between the real and predicted VA.

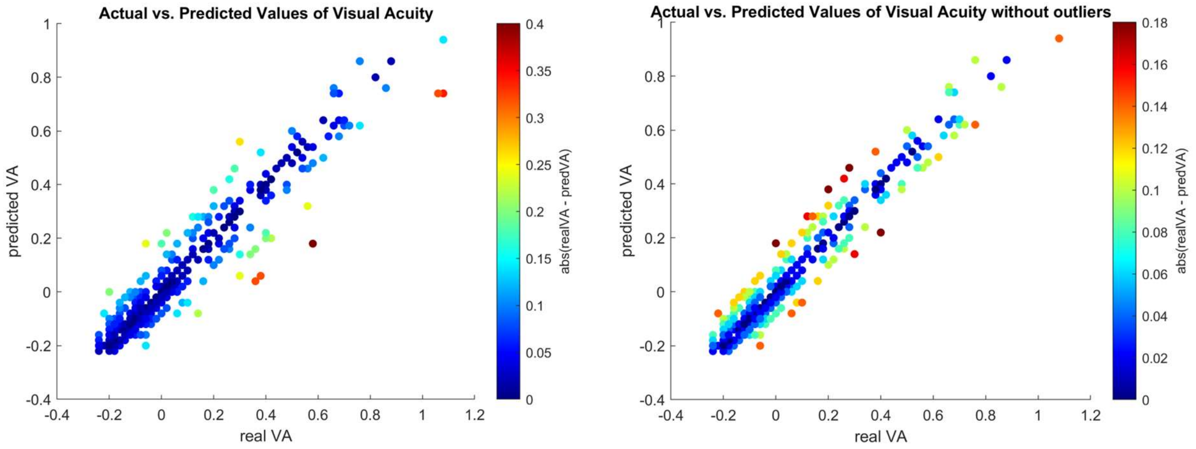

Figure S4. Plot showing the VA (in logMAR) as measured in the clinical trial (real VA) versus the XGBoost predicted VA (predicted VA) for the test set with (left) and without outliers (right). The color of the points is given by the absolute error between the real and predicted VA.

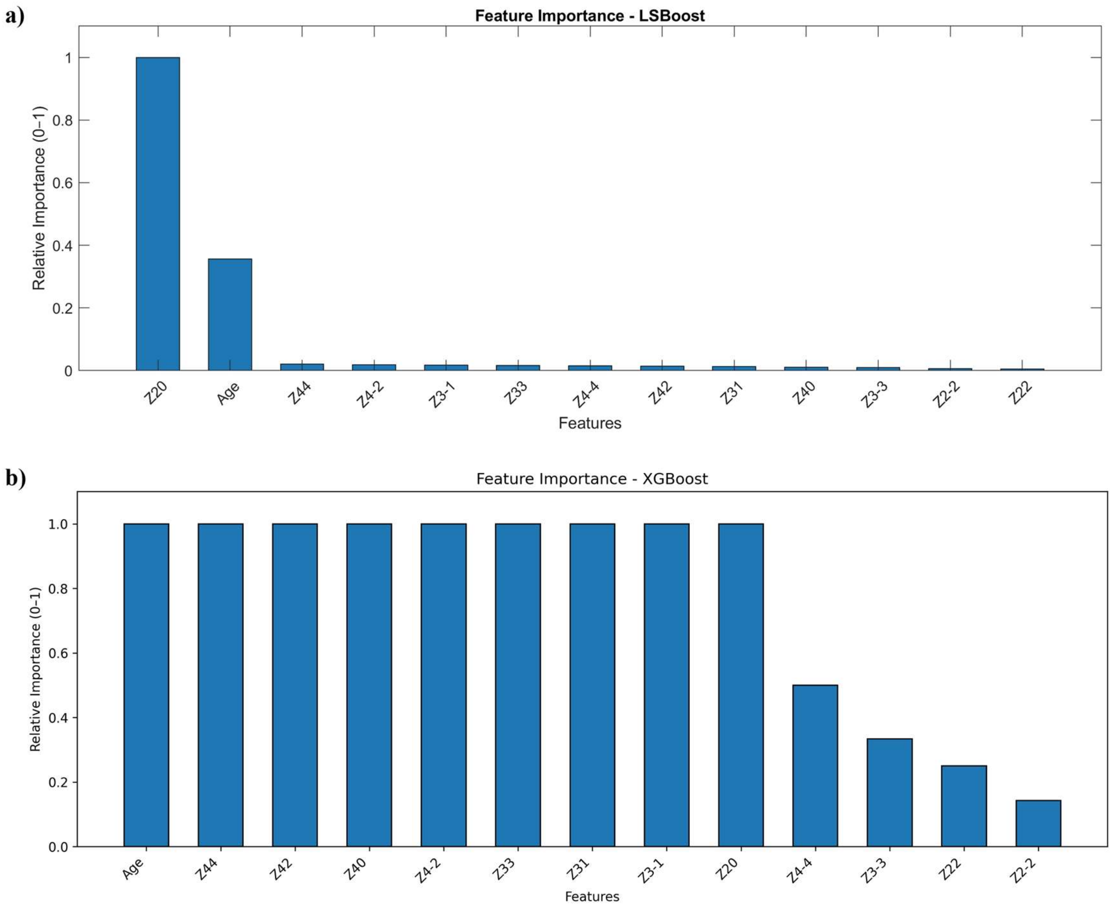

Figure S5. Feature Importance in LSBoost and XGBoost Models. Relative feature importance in LSBoost (a) and XGBoost (b) regression models. Each bar represents the normalized contribution (0–1) of each feature to the model's performance. Zernike polynomial features are denoted as Znm, where n is the radial degree and m the azimuthal degree.

(a) LSBoost 9 Zernikes and AA (with outliers)						
**Age group**	**30-40**	**41-44**	**45-50**	**51-55**	**56-60**	**61-65**
**Samples**	73	70	40	65	57	43
**MSE**	0.0057	0.0038	0.0084	0.0073	0.0056	0.0047
**RMSE**	0.0755	0.0613	0.0917	0.0853	0.0752	0.0687
**MAE**	0.0458	0.0483	0.0690	0.0658	0.0593	0.0535
**R^2**	0.8808	0.8983	0.8889	0.8795	0.9239	0.9407
**MaxE**	0.3400	0.1600	0.2600	0.3000	0.2000	0.2000
**MinE**	0.0000	0.0000	0.0000	0.0000	0.0000	0.0200
**Mode**	0.0000	0.0600	0.0600	0.0600	0.0000	0.0600
**Median**	0.0400	0.0400	0.0600	0.0600	0.0400	0.0400
**(b) LSBoost 9 Zernikes and AA (without outliers)**						
**MSE**	0.0017	0.0022	0.0046	0.0051	0.0037	0.0032
**RMSE**	0.0411	0.0474	0.0679	0.0716	0.0611	0.0564
**MAE**	0.0328	0.0397	0.0557	0.0584	0.0506	0.0468
**R^2**	0.9377	0.9341	0.9363	0.9131	0.9470	0.9509
**MaxE**	0.1000	0.1000	0.1600	0.1600	0.1200	0.1200
**MinE**	0.0000	0.0000	0.0000	0.0000	0.0000	0.0200
**Mode**	0.0000	0.0600	0.0600	0.0600	0.0000	0.0600
**Median**	0.0200	0.0400	0.0400	0.0600	0.0400	0.0400

Table S2. Metrics obtained with LSBoost (9 Zernike coefficients and amplitude of accommodation) stratified by age group in the test set. All metrics are expressed in logMAR, except for MSE, which is in logMAR^2, and R^2, which is dimensionless. Values are reported with (a) and without outliers (b), and the number of samples per age group is indicated.

(a) XGBoost 5 Zernikes (with outliers)						
**Age group**	**30-40**	**41-44**	**45-50**	**51-55**	**56-60**	**61-65**
**Samples**	73	70	40	65	57	43
**MSE**	0.0062	0.0042	0.0110	0.0094	0.0056	0.0073
**RMSE**	0.0790	0.0650	0.1050	0.0969	0.0746	0.0853
**MAE**	0.0501	0.0463	0.0720	0.0683	0.0600	0.0591
**R^2**	0.8693	0.8855	0.8542	0.8444	0.9250	0.9084
**MaxE**	0.3400	0.2200	0.3200	0.4000	0.2000	0.3200
**MinE**	0.0000	0.0000	0.0000	0.0000	0.0000	0.0000
**Mode**	0.0200	0.0200	0.0200	0.0200	0.0200	0.0200
**Median**	0.0400	0.0200	0.0400	0.0600	0.0400	0.0400
**(b) XGBoost 5 Zernikes (without outliers)**						
**MSE**	0.0026	0.0026	0.0074	0.0052	0.0040	0.0040
**RMSE**	0.0507	0.0511	0.0860	0.0724	0.0633	0.0629
**MAE**	0.0394	0.0394	0.0611	0.0571	0.0525	0.0488
**R^2**	0.9066	0.9199	0.8967	0.9110	0.9467	0.9388
**MaxE**	0.1400	0.1200	0.2000	0.1800	0.1200	0.1400
**MinE**	0.0000	0.0000	0.0000	0.0000	0.0000	0.0000
**Mode**	0.0200	0.0200	0.0200	0.0200	0.0200	0.0200
**Median**	0.0400	0.0200	0.0400	0.0600	0.0400	0.0400

Table S3. Metrics obtained with XGBoost (5 Zernike coefficients) stratified by age group in the test set. All metrics are expressed in logMAR, except for MSE, which is in logMAR^2, and R^2, which is dimensionless. Values are reported with (a) and without outliers (b), and the number of samples per age group is indicated.

	(a) Myopic (18 samples)			
	LSBoost		XGBoost	
	**With outliers**	**Without outliers**	**With outliers**	**Without outliers**
**MSE**	0.0195	0.0139	0.0201	0.0090
**RMSE**	0.1397	0.1177	0.1418	0.0949
**MAE**	0.1067	0.0929	0.1033	0.0750
**R^2**	0.8549	0.8855	0.8504	0.9141
**MaxE**	0.3400	0.2600	0.3400	0.2400
**MinE**	0.0000	0.0000	0.0000	0.0000
**Mode**	0.0000	0.0000	0.0200	0.0200
**Median**	0.0900	0.0800	0.0900	0.0700
	**(b) Emmetropic (26 samples)**			
	**LSBoost**		**LSBoost**	
	**With outliers**	**Without outliers**	**With outliers**	**Without outliers**
**MSE**	0.0041	0.0023	0.0031	0.0025
**RMSE**	0.0642	0.0480	0.0553	0.0498
**MAE**	0.0477	0.0383	0.0485	0.0442
**R^2**	0.6193	0.8013	0.7173	0.6639
**MaxE**	0.1800	0.1200	0.1000	0.0800
**MinE**	0.0000	0.0000	0.0000	0.0000
**Mode**	0.0000	0.0000	0.0200	0.0200
**Median**	0.0400	0.0400	0.0400	0.0400
	**(c) Hyperopic (9 samples)**			
	**LSBoost**		**LSBoost**	
	**With outliers**	**Without outliers**	**With outliers**	**Without outliers**
**MSE**	0.0306	0.0232	0.0360	0.0205

**RMSE**	0.1749	0.1522	0.1899	0.1434
**MAE**	0.1511	0.1325	0.1444	0.1125
**R^2**	0.4383	0.4830	0.3379	0.5410
**MaxE**	0.3000	0.2600	0.4000	0.3200
**MinE**	0.0200	0.0200	0.0200	0.0200
**Mode**	0.1000	0.1000	0.0200	0.0200
**Median**	0.1000	0.1000	0.1000	0.0900

Table S4. Metrics obtained with LSBoost and XGBoost stratified by refractive error group in the test set. All metrics are expressed in logMAR, except for MSE, which is in logMAR^2, and R^2, which is dimensionless. Values are reported with and without outliers, and the number of samples per group is indicated: (a) Myopic (SE < -0.5 D), (b) Emmetropic (-0.5 D $\leq$ SE $\leq$ 0.5 D), and (c) Hyperopic (SE $>$ 0.5 D). SE: spherical equivalent.

Model	Test set (subjects / observations)	RMSE (logMAR)
Watson & Ahumada [13]	4 subjects / 152 observations	0.056
LSBoost (this work)	20 subjects / 348 observations	0.058
XGBoost (this work)	20 subjects / 348 observations	0.063
Nestares et al. [12], verified by Dalimier et al. [21]	10 subjects / 140 observations	0.1 (decimal VA RMS = 0.23)
Füllep et al. [14]	8 subjects	0.045

Table S5. Comparison of functional visual acuity models, including the model type, test set size, and RMSE (logMAR).
